# Supplementary material for: Activation of the Chemokine Receptor CCR1 and Preferential Recruitment of Gαi Suppress RSV Replication: Implications for Developing Novel Respiratory Syncytial Virus Treatment Strategies
Source: J Virol. 2022 Nov 1;96(22):e01309-22. doi: 10.1128/jvi.01309-22 (PMC9682993; doi:10.1128/jvi.01309-22)
Supplement: Supplemental file 1 — Fig. S1. Download jvi.01309-22-s0001.pdf, PDF file, 0.6 MB [file jvi.01309-22-s0001.pdf]

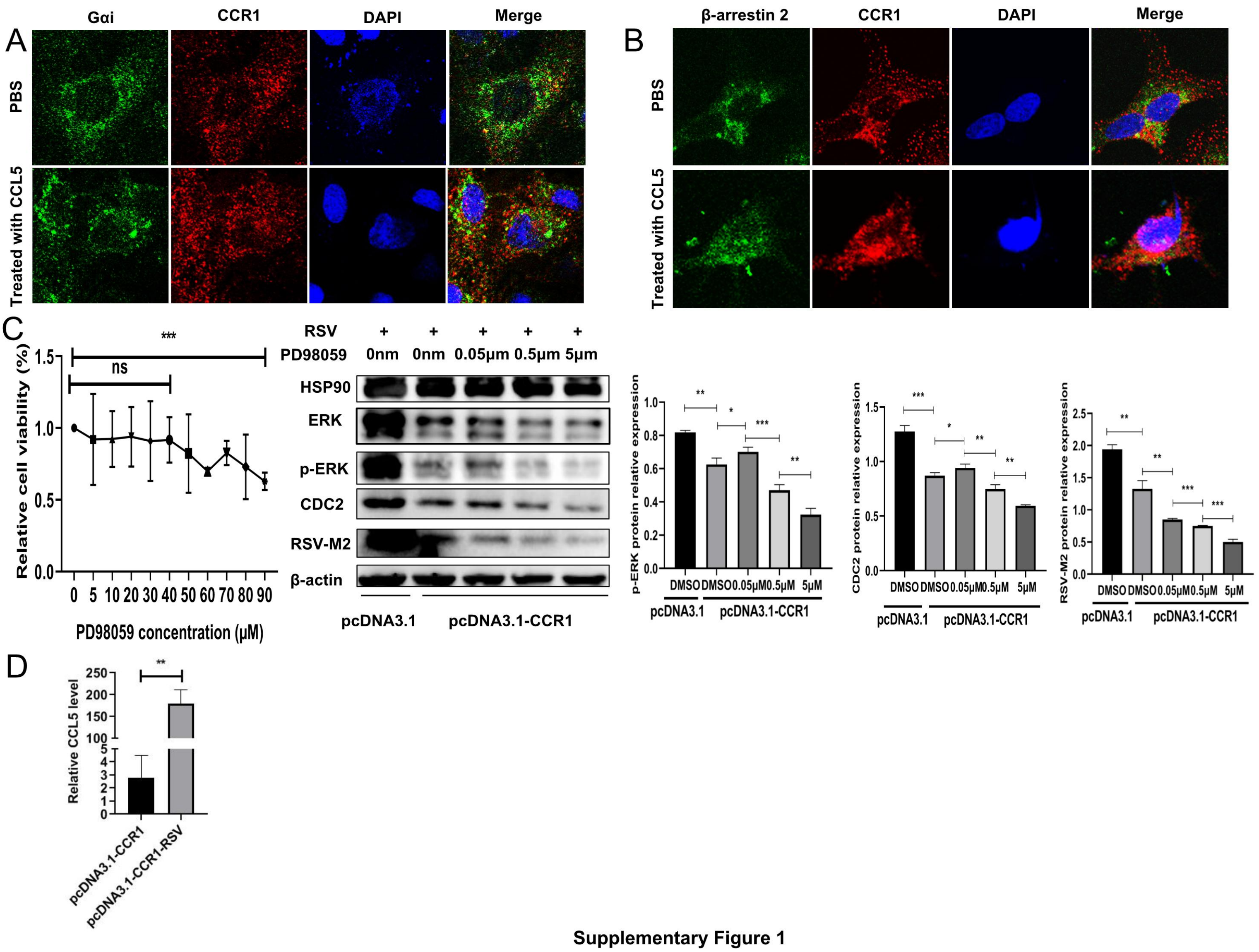

Supplementary Figure 1

Supplementary Fig. 1. (A, B) Following overexpression of CCR1 and Gαi or β-arrestin2 in A549 cells, the recruitment of Gαi or β-arrestin2 by CCR1 was observed by confocal microscopy. (C) CCR1-overexpressing A549 cells were pretreated with different concentrations of an ERK inhibitor (PD98059) for 30 minutes and then infected with RSV for 24h. The protein expression levels of CDC2, p-ERK and RSV-M2 were detected by Western blotting. (D)A549 cells were transfected with CCR1 plasmid for 30h, and then infected with RSV for 24 h, the expression of CCL5 was analyzed by qRT–PCR. (Unpaired t test, *P<0.05, **P<0.01, ***P<0.005, and ****P<0.001)
